# *Scedosporium* spp. from Clinical Setting in Argentina, with the Proposal of the New Pathogenic Species *Scedosporium americanum*

**DOI:** 10.3390/jof7030160

**Published:** 2021-02-24

**Authors:** Ruben A. Abrantes, Nicolás Refojo, Alejandra I. Hevia, Julián Fernández, Guillermina Isla, Susana Córdoba, María F. Dávalos, Silvina Lubovich, Ivana Maldonado, Graciela O. Davel, Alberto M. Stchigel

**Affiliations:** 1 Departamento Micología, Instituto Nacional de Enfermedades Infecciosas, Administración Nacional de Laboratorios e Institutos de Salud ‘Dr. C. G. Malbrán’, C1282 AFF Buenos Aires, Argentina; nrefojo@anlis.gob.ar (N.R.); ahevia@anlis.gob.ar (A.I.H.); jfernandez@anlis.gob.ar (J.F.); gisla@anlis.gob.ar (G.I.); scordoba@anlis.gob.ar (S.C.); gdavel@anlis.gob.ar (G.O.D.); 2 Hospital San Bernardo de la provincia de Salta, A4400 Salta, Argentina; flordavalos81@gmail.com; 3 Hospital Italiano de Buenos Aires, C1199 CABA Buenos Aires, Argentina; slubovich@gmail.com; 4 Hospital Alemán de Buenos Aires, C1118 AAT Buenos Aires, Argentina; ivanam27@gmail.com; 5 Mycology Unit, Faculty of Medicine and IISPV, Universitat Rovira i Virgili, 43201 Reus, Spain; albertomiguel.stchigel@urv.cat

**Keywords:** antifungal susceptibility, DNA sequencing, Microascales, phylogeny, *Scedosporium*

## Abstract

Species of the genus *Scedosporium* (family Microascaceae, phylum Ascomycota) are responsible for a wide range of opportunistic human infections, and have a low susceptibility to most antifungal drugs. It is well known that the pattern of *Scedosporium* species distribution varies according to geographic region. To assess the diversity of *Scedosporium* species in Argentina involved in human infections, we carried out a retrospective study reviewing 49 strains from clinical samples sent for diagnosis to the National Clinical Mycology Reference Laboratory between 1985 and 2019. Then, a phenotypic characterization, a phylogenetic study and and in vitro susceptibility test to antifungals were carried out. An analysis of combined nucleotide sequences dataset of the internal transcribed spacer of the ribosomal DNA (ITS) and of a fragment of the β-tubulin gene (*BT*2) demonstrated that 92% of the strains belonged to the species *S. boydii*, *S. apiospermum* and *S. angustum,* all them pertaining to *S. apiospermum* species complex. However, two strains (4%) were identified as *S. aurantiacum*, a species never reported in clinical settings in the Americas’. Surprisingly, one of them displayed a polycytella-like conidiogenesis, up to date only reported for *S. apiospermum*. In addition, the strain DMic 165285 was phylogenetically located far away from the rest of the species, so is proposed as the novel species *Scedosporium americanum*. On the other hand, from all seven antifungals tested, voriconazole and posaconazole were the most active drugs against *Scedosporium* spp.

## 1. Introduction

The Microascaceae is a monophyletic family of highly polymorphic fungi that includes the genera *Acaulium*, *Cephalotrichum*, *Fairmania*, *Fuscoannellis*, *Gamsia*, *Kernia*, *Lomentospora*, *Lophotrichus*, *Microascus*, *Parascedosporium*, *Petriella*, *Petriellopsis*, *Pithoascus*, *Pseudoscopulariopsis*, *Scedosporium*, *Scopulariopsis*, *Wardomyces*, *Wardomycopsis* and *Yunnania* [1,2,3]. Some of these genera are well-known human opportunistic human pathogens. The best known of these fungi is probably *Scopulariopsis brevicaulis*, a non-dermatophyte mostly causing onychomycosis. However, over the last two decades *Scedosporium* spp. have become increasing prevalent in immunocompromised patients, despite being better known for causing post-traumatic infections in healthy people [4]. The genus comprises species that are highly polymorphic: all them produce the scedosporium-like asexual morph, characterized by the production of solitary conidia from annelidic conidiogenous cells; a graphium-like synnematous synanamorph; unicellular sessile conidia; a polycytella-like synanamorph, a sort of “macroconidia” only seen in certain strains of *S. apiospermum* [5,6]; and a sexual morph, comprising dark, non-ostiolate globose ascomata with a thin peridial wall of textura epidermoidea, 8-spored, soon evanescent asci, and unicellular, yellowish or reddish brown, broadly fusiform to ellipsoidal ascospores with (usually) a germ pore at each end. The generic name was proposed by Saccardo (1911) [7], who described *Monosporium apiospermum* (isolated by Radaeli in 1911) from a case of eumycetoma) [8] that had morphological similarities with *Monosporium acremonioides* (actually *Harzia acremonioides*), the former producing non-verticillate, vaguely branched decumbent conidiophores (which are erect and verticillate in *H. acremonioides*) [9]. The generic name was validated by Castellani and Chalmers (1919), who accepted *Scedosporium apiospermum* instead of *M. apiospermum*. In 1943, Negroni and Fisher [10] isolated a fungus, which had asexual and sexual reproductive structures, from purulent material from a case of knee arthritis. Despite its great similarity to *Allescheria boydii* (isolated from a foot eumycetoma by Shear 1922) [11], they erected a new genus and species *Pseudallescheria shearii*. Consequently, the holomorph was given a different scientific name from the most common asexual morph (*Scedosporium*). In 2014, in order to remove the dual nomenclature that had been based on the anamorph/teleomorph concept, *Pseudallescheria* was accepted as a synonym of *Scedosporium* [12]. Ten species of *Scedosporium* are currently recognized (*S. aurantiacum*, *S. cereisporum*, *S. desertorum*, *S. dehoogii* and *S. minutisporum*, and *S. apiospermum* species complex including *S. angustum*, *S. apiospermum*, *S. boydii*, *S. ellipsoideum*, and *S. fusoideum*), which are easily distinguishable phylogenetically by comparing the sequences of a fragment of the β-tubulin gene (TUB2) rather than by phenotype [4,13]. *Scedosporium* species have been linked to various ecological niches, mostly being soils and waters contaminated with aliphatic and aromatic compounds, the consequence of anthropogenic industrial activity [14,15]. Infections by *Scedosporium* spp. cause a wide range of clinical entities, such as osteomyelitis, keratitis, lymphocutaneous infection and sinusitis [4,16,17,18] and is the primary etiological agent of white grain mycetoma worldwide [19]. *Scedosporium* spp. can also colonize the respiratory tract of cystic fibrosis patients (CF), causing allergic bronchitis, and has produced disseminated diseases and central nervous system (CNS) infections in immunocompetent patients after near-drowning incidents. *Scedosporium* spp. infections occur by traumatic implantation of contaminated materials (e.g., wood chips), or can be acquired by inhalation of contaminated aerosols [16,17].

The most common systemic antifungals display high minimum inhibitory concentration (MIC) values for *Scedosporium* spp., making successful treatment difficult. The main clinically relevant species, *S. apiospermum* and *S. boydii*, show variable, strain-dependent in vitro antifungal susceptibility [20,21]. Another clinically important species, *S. aurantiacum*, also has high MIC values against every antifungal apart from voriconazole (VZ), which is the first-line antimycotic drug against *Scedosporium* spp. infections [21].

Several reports show that *S. apiospermum* species complex is found worldwide [4]. However, *Scedosporium aurantiacum* has been reported to colonise airways of patients primarily in Europe and Australia, where there is a larger number of patients with CF [22,23,24]. It has also been isolated once in Japan [25] but so far has not been reported in clinical cases in the Americas.

The aim of this work is to increase knowledge of the diversity of *Scedosporium* spp. involved in human infections in Argentina by presenting the results of a retrospective study of 49 clinical isolates that includes phylogenetic analyses, phenotypic characterizations and in vitro susceptibility assays to antifungals. *Scedosporium aurantiacum* and *S. dehoogii* are reported for the first time in clinical samples for the Americas, and *Scedosporium americanum* is described as a novel species.

## 2. Materials and Methods

### 2.1. Fungal Strains

We studied 49 fungal strains that were received for identification between 1985 and 2019 at the laboratory of the Mycology Department of the National Institute of Infectious Diseases, ‘Dr. Carlos G. Malbrán’. The strains were sent from public and private hospitals of eight Argentinian provinces (Table 1).

### 2.2. DNA Extraction, Amplification and Sequencing

Total DNA was extracted and purified from fungal colonies according to Refojo et al. [26]. The internal transcribed spacer (ITS) region of the nuclear ribosomal DNA and a fragment of the β-tubulin (*BT*2) gene were amplified with the primers ITS5 and ITS4 and Bt2a and Bt2b, respectively [27,28]. The PCR reaction mixture (50 μL) included 10 to 20 ng of fungal DNA template, MgCl2 1.5 mM, dNTPs 250 mM, TRIS-HCl 20 mM (pH 8.4), KCl 50 mM, 2.5 U Taq DNA polymerase (Invitrogen, Life Technologies, Carlsbad, CA, USA) and 0.2 μM of each primer. The amplification programme included an initial denaturation step at 94 °C for 5 min followed by 35 cycles of denaturation at 95 °C for 45 s, annealing for 1 min at 55 °C (ITS) or 59 °C (BT2), and extension for 1 min at 72 °C. A final extension step at 72 °C for 10 min was added at the end of the amplification. After PCR, the products were purified with Accuprep^®^ PCR Purification Kit (Bioneer, Oakland, CA, USA) and stored at −20 °C until they were used. PCR products were sequenced in both directions using the primers described above, using the Big Dye ™ Terminator v3.1 Cycle Sequencing kit (Applied Biosystems, Foster City, CA, USA) according to the manufacturer’s instructions, on a 3500 ABI genetic analyzer (Applied Biosystems). The sequences obtained were edited using Bioedit Sequence Alignment Editor v7.0.4.1 [29]. 

### 2.3. Molecular Identification and Phylogeny

A preliminary identification was carried out using the Basic Local Alignment Search Tool (BLAST; https://blast.ncbi.nlm.nih.gov/Blast.cgi). For species-level identification, an identity ≥ 98% (and a coverage ≥99%) was considered when the nucleotide sequences of our strains were compared with those of type or reference strains. ITS and BT2 sequences were aligned using Muscle in MEGA v6.0 software followed by manual adjustment. The best-fit model of sequence evolution was determined by MODELTEST v2.3 [30,31]. For phylogenetic analysis, sequences of ex-type strains of *Scedosporium* spp. and related taxa of the Microascaceae were taken from GenBank (https://www.ncbi.nlm.nih.gov/genbank/). All sequences generated in this study were deposited in GenBank and their accession numbers are listed in Table 1. Gene sequences of *Parascedosporium tectoneae* (CBS 120338) were used as outgroup. The computer program MEGA v6.0 [30] was used to build the phylogenetic tree based on the maximum-likelihood (ML) algorithm with a Kimura-2-parameters model with a gamma distribution, treating gaps as complete deletions. A 1000 bootstrap replicates was used, and bootstrap support (BS) values ≥ 75% were considered significant. Other phylogenetic analyses were carried out by MrBayes v3.1.2 [32]. Two parallel runs of four chains were performed for 10,000,000 generations, and trees were sampled every 100 generations. TRACER version 1.5 was used to verify that the mean likelihood value, effective sample size (ESS), and other parameters reached a plateau. For each run, 25% of the trees were discarded as they were obtained during the burning phase. The posterior probability (PP) is shown with a cut-off of 0.95. Trees were viewed and edited with FIGTREE v1.1.2 (http://tree.bio.ed.ac.uk/software/figtree/) and MEGA v6.0 [30] software.

### 2.4. Morphological Characterization

The strains were cultured at 25, 37, 40, and 45 °C in the dark onto potato dextrose agar (PDA; 75 g potatoes, 20 g dextrose, 15 g agar-agar, 1 L tap water; home made) and oatmeal agar (OA; 30 g oat flakes, 1 g MgSO4·7H2O, 1.5 g H2PO4, 15 g agar, 1 L tap water; home made). Also, the fungal strains were grown at 15, 38, 39, 42 and 43 °C when it was considered necessary to gather such information. Cultures were characterized after 7 days and 14 days of incubation, color notations in parentheses are from Kornerup and Wanscher [33], reproductive structures were observed from slide cultures using 65% lactic acid under a Leica DMF2500 (Leica Microsystems, Concord, ON, Canada) bright field microscope. The measurements of fungal structures and the micrographs were taken by a Leica DMC 2900 camera with LAS v4.8 software (Leica Microsystems, Concord, ON, Canada).

### 2.5. Antifungal Susceptibility Testing

MIC values were determined according to the reference document M38-3rd ed. from the Clinical and Laboratory Standards Institute (CLSI) [34]. The antifungal drugs tested were: amphotericin (AMB), itraconazole (ITR) and voriconazole (VOR) (Sigma-Aldrich, Buenos Aires, Argentina), caspofungin (CAS) and posaconazole (POS) (Merck, Co., Argentina), anidulafungin (ANI) (Pfizer, Buenos Aires, Argentina) and micafungin (MICA) (Astellas, Tokio, Japan). The strains were cultured on PDA at 35 °C for 5–7 days to induce sporulation. Because strains DMic 165285 and CBS 218.35 showed little growth on PDA, both were cultured on OA. For AMB and triazoles, the MIC endpoint was considered at 100% of growth inhibition, while for echinocandins the minimal effective concentration (MEC) was used, which is the concentration of drug at which hyphae are rounded and compact, and stopped growing (CLSI M38) when compared with the growth control well. Geometric mean, mode, range, MIC50 and MIC90 were calculated. Neither clinical breakpoint nor epidemiological cut-off values have been defined for *Scedosporium* spp., therefore the susceptible/resistant and wild type/not-wild type categories were not used. For analytical purposes, we assigned a cut-off MIC value ≥2 mg/L to interpret results. *Candida parapsilosis* ATCC 22019, *C. krusei* ATCC 6258 and *Aspergillus flavus* ATCC 204304 were used as controls in all assays.

## 3. Results

### 3.1. Molecular Presumptive Identification of the Strains

A BLAST search using ITS sequences identified most of our fungal strains as *S. boydii* (33/49 strains), followed by *S. apiospermum* (12/49), *S. aurantiacum* (2/49), and *S. dehogii* and *S. ellipsoideum* (one strain each). However, when BLAST was performed using *TUB*2 sequences, five of the strains re-identified as *S. angustum*, and two others as *S. ellipsoideum* (Tables S1 and S2). On the other hand, one of our strains (DMic 165285) identified as *S. boydii* by ITS BLAST could not be located at species level using TUB2 sequence.

### 3.2. Phylogenetic Analyses

**Figure 1:**
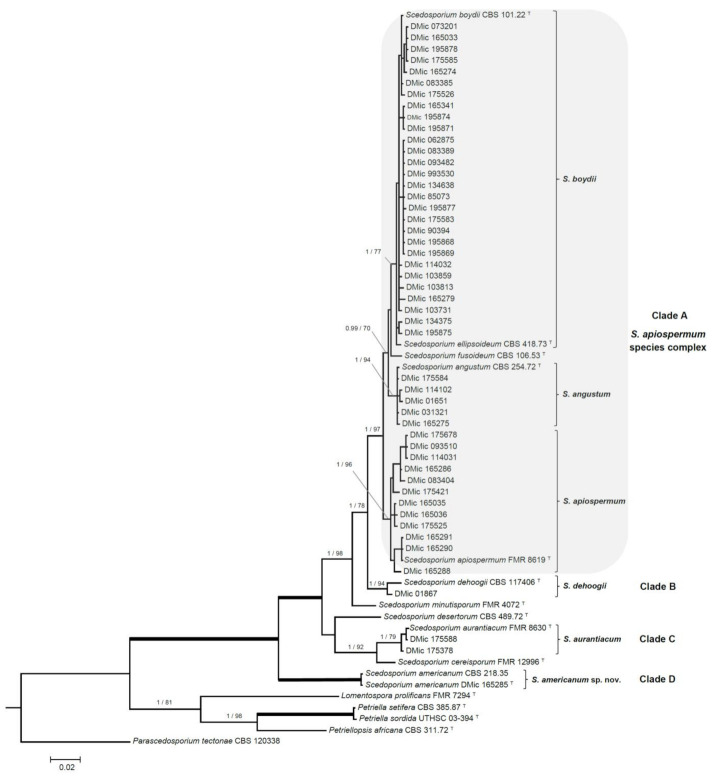
Bayesian phylogenetic tree built using DNA sequences from two *loci* (ITS and *BT*2). Bayesian posterior probability values over 0.95 and bootstrap support scores over 70 % are indicated on the nodes. Branches in bold represent 1/100 values. *Parascedosporium tectonae* was selected as outgroup. ^T^ = ex-type strain. Alignment length 989 bp. The sequences not generated by us were retrieved from GenBank.

The phylogenetic study included 65 ITS and TUB2 sequences, with a total of 989 characters including gaps, from which 244 were parsimony informative. The ML analysis was congruent with the BI analysis, both displaying a similar topology. In the combined ITS-*TUB*2 tree, our strains were distributed across four clades, into a well-supported main clade (1 PP/100 BS) grouping all *Scedosporium* species (Figure 1). Forty-five of the isolates (92%, 45/49) were placed into the *S. apiospermum* species complex clade A (1 PP/97% BS), and distributed across several terminal clades: A terminal clade (1 PP/77% BS) containing the type strains of *S. boydii* and *S. ellipsoideum*, included 28 of our strains, displaying a relatively high intraspecific genetic variation; another twelve strains were placed into a terminal clade (1 PP/96% BS) containing the type strain of *S. apiospermum*, also with a relatively high intraspecific genetic variation, and a third terminal clade (1 PP/94% BS) included the type strain of *S. angustum* and four of our strains. Clade B (1 PP/94% BS) included only the type strain of *S. dehoogii* and one of our strains. Clade C (1 PP/79% BS) included two Argentinian strains and the type strain of *S. aurantiacum* (Figure 1, Table 1). And clade D (1 PP/100% BS), placed in a basal position and phylogenetically distant from the rest of *Scedosporium* spp., included the strain DMic 165285 and CBS 218.35. The latter strain was included in the phylogeny because it displayed the highest identity in the BLAST search (ITS: 546/551 bp, 99.1%, 5 gaps). The estimation of the evolutionary distance showed a greater proximity within *Scedosporium* spp. than those from other related genera belonging to the Microascaceae (data not shown).

### 3.3. Morphology

**Figure 2:**
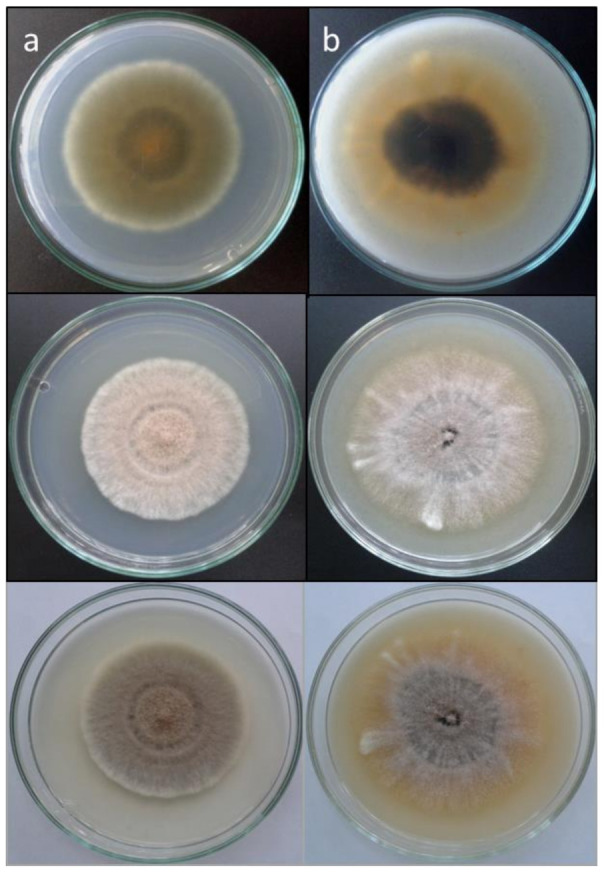
*Scedosporium aurantiacum* DMic 175378. (**a**) Colony on PDA (column, from top to down: reverse and surface on a black background, and surface on a white background) after 14 days at 25 °C. (**b**) Colony on OA (same explanation than previously) after 14 days at 25 °C.

**Figure 3:**
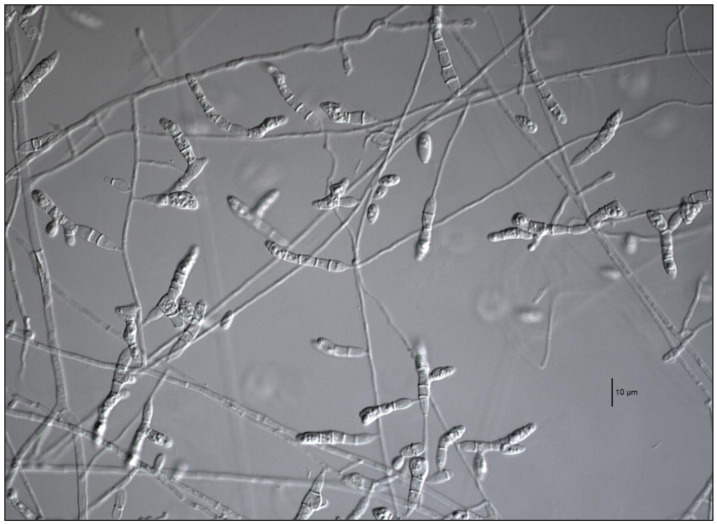
*Scedosporium aurantiacum* DMic 175588. Polycytella-like asexual morph. Bar = 10 µm.

All strains formed the typical scedosporium-like asexual morph, and were able to grow up to 37 °C. Also, 42/45 strains of the *S. apiospermum* species complex (clade A) were able to grow up to 40 °C. Three of our strains that failed to grow at 40 °C were in the *S. boydii*/*S. ellipsoideum* terminal clade. In clade A, 23 of our strains produced ascomata on PDA and OA, 18 corresponding to *S. boydii* and 5 to *S. angustum*. None of our *S. apiospermum* strains formed ascomata in either culture media tested. Strain DMic 01867, placed in clade B (*S. dehoogii*), did not produce a synnematous asexual morph or a sexual morph, nor grew at 40 °C. Two of the strains placed in clade C (*S. aurantiacum*) produced the characteristic yellowish pigment in both culture media tested (Figure 2), did not manifest the sexual morph either, but both produced the synnematous asexual morph [35], and grew up to 42 °C, despite it being described that some strains can grow up to 45 °C. Strain DMic 175588 formed, in addition, a polycytella-like asexual morph (Figure 3), so far only reported for *S. apiospermum* [6]. Strains DMic 165285 and CBS 218.35, in the clade D (not matching any of previously known species of the genus) failed to grow at 40 °C, and their growing rates on OA and PDA at 25 °C and at 37 °C were significantly lower than the rest of the species [36] (Table S3).

### 3.4. Taxonomy

Given the phylogenetic distance between clade D and the rest of those representing the species of *Scedosporium*, and due to the distinctiveness of their phenotype, we erect *Scedosporium americanum*, selecting DMic 165285 as its ex-type strain, due to the greatest diversity of reproductive structures.

**Figure 4:**
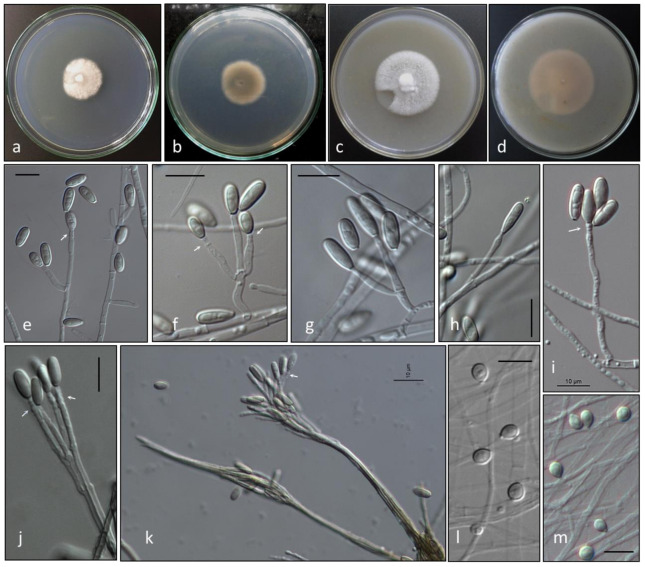
*Scedosporium americanum* DMic 165285 ^T^. Colony on PDA (**a**, surface; **b**, reverse), and on OA (**c**, surface; d, reverse) after 14 days at 25 °C. Conidiogenous cells and conidia (**e**–**i**). Synnematous conidiomata. (**j**,**k**). Sessile conidia (**l**,**m**). Arrows indicate annellations at the top of conidiogenous cells. Bars = 10 µm.

**Figure 5:**
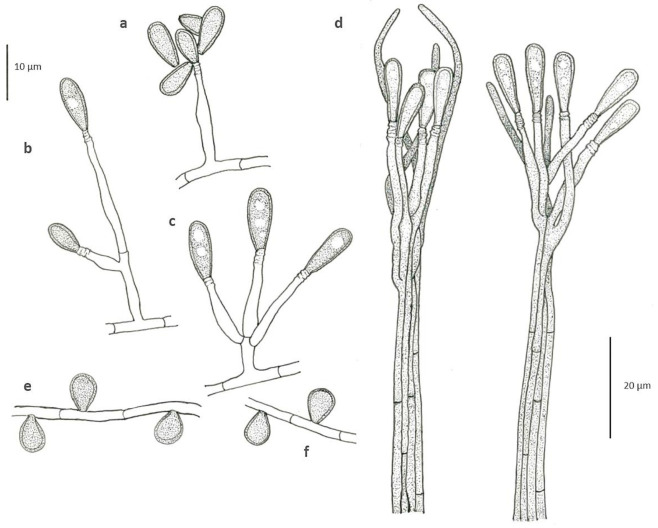
*Scedosporium americanum* DMic 165285 ^T^. Conidiogenous cells and conidia (**a**–**c**). Synnematous conidiomata. (**d**). Sessile conidia (**e**,**f**).

*Scedosporium americanum* Abrantes, Refojo, Hevia, J. Fernández et Stchigel, sp. nov. (Figure 4 and Figure 5). MycoBank MB 836850.

**Etymology:** Due to the geographical origin of the fungus.

**Typus: Argentina**, Salta, from hand subcutaneous lesions of a man, 5 Febr. 2016, M. F. Dávalos (holotype DMic-H 165285, culture ex-type DMic 165285).

**Additional material examined:** USA, unknown origin (cited as probably mycetoma pedis), April 1935, Fred D. Weidmann (culture CBS 218.35).

*Mycelium* composed by septate, hyaline to pale brown, smooth-, thin- to thick-walled hyphae of 1.3–2.9 μm wide. *Conidiophores* solitary, often reduced to a single conidiogenous cell arising laterally from undifferentiated hypha, or short-stalked (5–15 µm long), bearing two or three conidiogenous cells at the top. *Conidiogenous cells* annellidic, lateral or terminal, hyaline, smooth- and thin-walled, cylindrical to flask-shaped, 5–16 µm long, 1.5–2.5 µm wide, with several, distinct annellations at the top with the age. *Conidia* enteroblastic, one-celled, solitary, arranged in slimy masses, hyaline to sub-hyaline, ovoid when young, 7.5–8.8 × 3.8–4.5 μm, then becoming brown, smooth- and thick-walled, gutulate, ellipsoidal to claviform with a truncated base, 10–13.6 × 3.1–4.8 μm. *Conidiomata* synnematous, more abundantly produced on PDA after 4 weeks at 25 °C, consisting in 3–5 adpressed elongated conidiophores, attaining up to 210 μm in length at first determined, then increasing its length with age; conidiogenous cells annellidic, indistinguishable from those of the conidiophores; conidia claviform with a truncate base, 11–17 × 3.3–3.8 μm. *Sessile conidia* holoblastic, one-celled, solitary, scattered and scarcely produced, ovoid, 5.4–6.8 × 4.2–4.8 μm, attached directly to the hyphae or on short lateral branches, more abundant on OA than on PDA. *Sexual morph* not observed.

Colonies on PDA attaining 30–32 mm diam after 14 days at 25 °C, cottony, slightly elevated, woolly at centre, nearly white, margins irregular and fimbriated; reverse brown at centre (4D2) and olive brown (4E8) to colourless towards the periphery. Colonies on OA attaining 40–45 mm diam after 14 d at 25 °C, slightly elevated at the centre, woolly to floccose, white, with slightly greyish patches (4C1), margins lobed; reverse yellowish grey (4B2) at the centre and greyish to colourless towards the periphery. Colonies on PDA and OA at 37 °C after 7 days attaining 5–6 mm and 9–10 mm, respectively. The fungus does not produce diffusible pigment on PDA or OA. The minimum, optimum and maximum temperatures of growth are: 15 °C, 25–30 °C and 39 °C, respectively.

**Figure 6:**
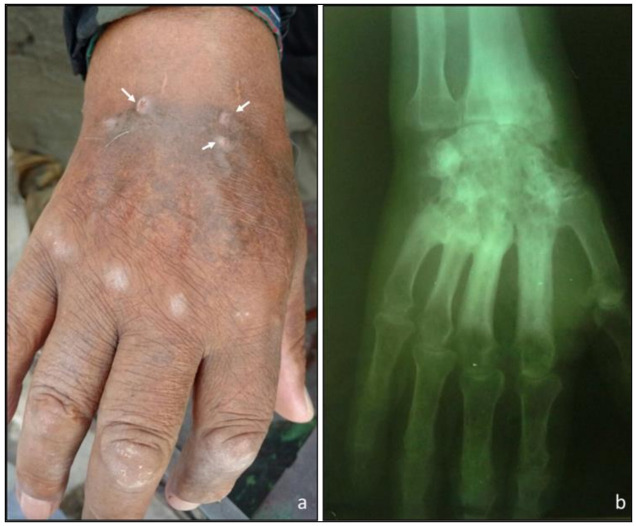
Subcutaneous infection of the right hand: arrows indicate drain sinuses produced in the lesion (**a**). X-ray of hand showing bone lesions (**b**).

**Case report:** DMic 165285 was recovered in 2016 from a 76-year-old man who lives in Salta city, a blacksmith by trade, with type II diabetes and no other previous history of disease. He reported not remembering any major trauma, only small skin punctures by metal shavings. He had a hand lesion of 18 years of evolution, and presented fistulae draining pus and local bone involvement. The fungus was recovered from two clinical samples (a biopsy of subcutaneous tissues and a pus discharge from a fistulated tract) by culturing on Sabouraud’s dextrose agar. On direct examination with KOH 30%, only septate hyaline hyphae were seen, and the drainage material did not contain eumycotic grains (Figure 6). Unfortunately, the patient did not return for medical consultation, so it was not possible to follow its clinical evolution.

### 3.5. Antifungal Susceptibility Testing

The MIC and MEC values obtained are given in Table 2. Overall, 66% of the tested strains (33/50) showed a MIC value ≥2 mg/L for AMB, followed by 54% (27/50) for ANI, 50% (25/50) for CAS, 28% (14/50) for ITR, and 16% (8/50) for MICA. However, VOR and POS were the most active antifungals, and only two isolates exhibited MIC values ≥2 mg/L for either drug.

## 4. Discussion

Our results demonstrate that the most frequently recovered species of *Scedosporium* from clinical specimens in Argentina belonged to the *S. apiospermum* species complex (clade A; Figure 1), which is clearly monophyletic and presents an intraspecific genetic variability consistent with its definition [13]. This variation is mainly manifested in the *S. boydii* and *S. apiospermum* subclades, as described in the review by Ramírez-García et al. [4]. In our phylogenetic analysis, clade A was divided into two robust subclades, where the homothallic lineages (corresponding to *S. boydii* and to *S. angustum*) are separated from the heterothallic ones (*S. apiospermum*). *Scedosporium aurantiacum* (clade B) is represented by two strains from sputum of patients with CF. The strain DMic 175378 was from an 11-year-old patient (already deceased) from Santa Fé province, who never left the country, so we consider this as the first recorded autochthonous case of infection by *S. aurantiacum*. The second strain (DMic 175588) was recovered from a 27-year-old diabetic patient in Buenos Aires city, who had a history of travelling to Egypt and Europe [14]. It is remarkable that this isolate displays a polycytella-like asexual morph previously reported only in an “aberrant” strain of *S. apiospermum* [5,6]. Both cases of infection by *S. aurantiacum* represent the first report in the Americas. Furthermore, we report for the first time an autochthonous case of infection by *S. dehoogii*, although this fungus has been isolated from environment in Chile and Mexico [37,38]. Surprisingly, our strain DMic 165285 was found to represent a novel species, *Scedosporium americanum* (clade D). In 2008, Gilgado et al. [39] carried out a comprehensive study of the genus *Scedosporium* and its sexual counterpart, *Pseudallescheria*. These authors included the strain CBS 218.35, highlighting that, despite it showing morphological similarities with the asexual reproductive structures of other *Scedosporium* spp., it was phylogenetically placed at distance from the other species studied. This suggested that further studies were required to determine whether or not it is a different species, which we believe has been resolved during the development of our work. We have erected the novel species *Scedosporium americanum* since it produces a greater diversity of reproductive structures and because CBS 218.35 has a dubious geographical and clinical origin, and we have chosen DMic-H 165285 as its type material.

In the present study, MIC values of antifungals tested against *S. boydii*, *S. apiospermum* and *S. angustum* are in agreement with those reported by other authors [21,37,40,41]. In addition, we have confirmed VOR and POS show the highest activity in vitro against these species (MIC values ≤1 mg/L against 48/50 strains) and that the in vitro behaviour of antifungal drugs against the fungi is intra- and interspecies-dependent. Interestingly, among echinocandins, micafungin was slightly more active against all *Scedosporium* strains tested, our results being in agreement with those of others researchers [20,37].

In conclusion, the present study demonstrates the diversity of *Scedosporium* species from clinical samples in Argentina, expanding the currently available epidemiological information. Despite there being an increasing number of reports of infections due to *Scedosporium* spp. [18] in recent years, their identification is, in many countries, based on conventional morphological characterization and mostly limited at genus-level. We highlight the importance of supporting continuous scrutiny of not only possible changes in distribution of the species but also in the emergence of other species that are poorly or not susceptible to the most common antifungal drugs.

## Figures and Tables

**Table 1:** Strains and sequences used in the study.

Taxon	Collection Accession Number	Source	GenBank Accession Number	
			*BT*2	ITS
*Scedosporium boydii*	DMic 85073	No data, 1985	**MT813143**	**MT803004**
*Scedosporium boydii*	DMic 90394	No data, 1990	**MT813144**	**MT803005**
*Scedosporium boydii*	DMic 993530	Lung tissue, 1999	**MT813145**	**MT803006**
*Scedosporium angustum*	DMic 01651	No data, 2001	**MT813146**	**MT803007**
*Scedosporium dehoogii*	DMic 01867	Corneal abscess, 2001 *	**MT813147**	**MT803008**
*Scedosporium angustum*	DMic 031321	No data, 2003	**MT813148**	**MT803009**
*Scedosporium boydii*	DMic 062875	Lung tissue, 2006	**MT813149**	**MT803010**
*Scedosporium boydii*	DMic 073201	Sputum, 2007	**MT813150**	**MT803011**
*Scedosporium boydii*	DMic 083385	Sputum, 2008	**MT813151**	**MT803012**
*Scedosporium boydii*	DMic 083389	Lung tissue, 2008	**MT813152**	**MT803013**
*Scedosporium apiospermum*	DMic 083404	Corneal abscess, 2008	**MT813153**	**MT803014**
*Scedosporium boydii*	DMic 093482	Lung tissue, 2009	**MT813154**	**MT803015**
*Scedosporium apiospermum*	DMic 093510	Corneal abscess, 2009	**MT813155**	**MT803016**
*Scedosporium boydii*	DMic 103859	Sputum, 2010	**MT813156**	**MT803017**
*Scedosporium boydii*	DMic 103731	Corneal abscess, 2010	**MT813157**	**MT803018**
*Scedosporium boydii*	DMic 103813	Corneal abscess, 2010	**MT813158**	**MT803019**
*Scedosporium apiospermum*	DMic 114031	Corneal abscess, 2011	**MT813159**	**MT803020**
*Scedosporium boydii*	DMic 114032	Bronchoalveolar lavage, 2011	**MT813160**	**MT803021**
*Scedosporium angustum*	DMic 114102	Subcutaneous mycosis (foot), 2011	**MT813161**	**MT803022**
*Scedosporium boydii*	DMic 134638	Sputum, 2013	**MT813162**	**MT803023**
*Scedosporium boydii*	DMic 165274	Subcutaneous mycosis (leg), 2013	**MT813163**	**MT803024**
*Scedosporium boydii*	DMic 134375	Sputum, 2014	**MT813164**	**MT803025**
*Scedosporium angustum*	DMic 165275	Corneal abscess, 2014	**MT813165**	**MT803026**
*Scedosporium boydii*	DMic 165279	Tracheal aspirate, 2014	**MT813166**	**MT803027**
*Scedosporium boydii*	DMic 165033	Bronchoalveolar lavage, 2015	**MT813167**	**MT803028**
*Scedosporium apiospermum*	DMic 165035	Subcutaneous mycosis (arm), 2015	**MT813168**	**MT803029**
*Scedosporium apiospermum*	DMic 165036	Corneal abscess, 2015	**MT813169**	**MT803030**
*Scedosporium apiospermum*	DMic 165286	Subcutaneous mycosis (hand), 2015	**MT813170**	**MT803031**
*Scedosporium americanum* sp. nov.	DMic 165285	Subcutaneous mycosis (hand), 2016	**MT813171**	**MT803032**
*Scedosporium apiospermum*	DMic 165288	Bronchoalveolar lavage, 2016	**MT813172**	**MT803033**
*Scedosporium apiospermum*	DMic 165290	Bronchoalveolar lavage, 2016	**MT813173**	**MT803034**
*Scedosporium apiospermum*	DMic 165291	Brain biopsy, 2016	**MT813174**	**MT803035**
*Scedosporium boydii*	DMic 165341	Sputum, 2016	**MT813175**	**MT803036**
*Scedosporium aurantiacum*	DMic 175378	Sputum, 2016	**MT813176**	**MT803037**
*Scedosporium apiospermum*	DMic 175421	Subcutaneous mycosis, turtle shell **, 2016	**MT813177**	**MT803038**
*Scedosporium apiospermum*	DMic 175525	Sputum, 2017	**MT813178**	**MT803039**
*Scedosporium boydii*	DMic 175526	Sputum, 2017	**MT813179**	**MT803040**
*Scedosporium boydii*	DMic 175583	Sputum, 2017	**MT813180**	**MT803041**
*Scedosporium angustum*	DMic 175584	Sputum, 2017	**MT813181**	**MT803042**
*Scedosporium boydii*	DMic 175585	Brain biopsy, 2017	**MT813182**	**MT803043**
*Scedosporium aurantiacum*	DMic 175588	Sputum, 2017	**MT813183**	**MT803044**
*Scedosporium boydii*	DMic 195868	Sputum, 2017	**MT813184**	**MT803045**
*Scedosporium apiospermum*	DMic 175678	Tracheal aspirate, 2017	**MT813185**	**MT803046**
*Scedosporium boydii*	DMic 195875	Bronchoalveolar lavage, 2018	**MT813186**	**MT803047**
*Scedosporium boydii*	DMic 195871	Bx cutanea, 2018	**MT813187**	**MT803048**
*Scedosporium boydii*	DMic 195874	Sputum, 2018	**MT813188**	**MT803049**
*Scedosporium boydii*	DMic 195869	Sputum, 2018	**MT813189**	**MT803050**
*Scedosporium boydii*	DMic 195877	Sputum, 2018	**MT813190**	**MT803051**
*Scedosporium boydii*	DMic 195878	Cerebrospinal fluid, 2018	**MT813191**	**MT803052**
*Scedosporium* sp.	CBS 218.35	Human mycetoma pedis, Paraguay	**MT813192**	AM712309
*Parascedosporium tectonae*	CBS 120.338	Garden soil Buenos Aires, Argentina	AM409105	AM409113
*Petriellopsis africana*	CBS 311.72	Brown sandy soil, Namibia	AJ889603	AJ888425
*Scedosporium angustum*	CBS 254.72	Half-digested-sewage tank Ohio, USA	AJ889604	AJ888414
*Scedosporium boydii*	CBS 101.22	Mycetoma Texas, USA	AJ889590	AJ888435
*Scedosporium desertorum*	CBS 489.72	Salt-marsh soil, Kuwait	AM409106	AM409101
*Scedosporium ellipsoideum*	CBS 418.73	Soil, Tayikistán	AJ889595	AJ888426
*Scedosporium fusoideum*	CBS 106.53	Goat dung Aligarh, India	AJ889601	AJ888428
*Scedosporium apiospermum*	FMR 8619	Keratitis, Brazil	AJ889584	NR130664
*Scedosporium minutisporum*	FMR 4072	River sediment Tordera river, Spain	AJ889592	AJ888384
*Scedosporium aurantiacum*	FMR 8630	Ankle ulcer S. Compostela, Spain	AJ889597	AJ888440
*Scedosporium dehoogii*	CBS 117406	Garden soil, Barcelona, Spain	KT163401	KT163400
*Scedosporium cereisporum*	FMR 12996	Wastewater sludge, Mûrs-Erignés, France	KJ599659	KJ599660
*Lomentospora prolificans*	FMR 7294	Clinical California, USA	AJ889591	AJ888444
*Petriella setifera*	CBS 385.87	Human toe nail Helsinki, Finland	EU977491	AY882345
*Petriella sordida*	UTHSC 03-394	Nasal infection California, USA	AM409104	AM409114

* Strain sent by the Department of Microbiology of the National University of Asunción, Paraguay, to the National Reference Laboratory for Clinical Mycology of the National Institute of Infectious Diseases–ANLIS ‘Dr. Carlos G. Malbrán’. ** *Chelonia mydas*. CBS: Culture collection of the Westerdijk Biodiversity Institute, Utrecht, the Netherlands; DMic: Culture collection of fungi of biomedical interest (World Data Centre for Microorganisms # 1115), Buenos Aires, Argentina; FMR: Faculty of Medicine Reus culture collection, Spain; UTHSC: Collection of the Fungus Testing Laboratory, University of Texas Health Science Center at San Antonio, USA. The sequence access numbers generated in this study are indicated in **bold**. ITS: internal transcribed spacer region 1 and 2 including 5.8S rDNA; *BT*2: β-tubulin protein-coding gene.

**Table 2:** MIC and MEC values to seven antifungal drugs against *Scedosporium* spp.

Strains	MIC Parameters	Antifungal Drugs (mg/L)						
		AMB	ITR	VOR	POS	ANI *	CAS *	MICA *
*S. boydii* (*n* = 28)	GM	1.52	0.34	0.15	0.16	0.53	0.44	0.19
	MIC 50	2	0.5	0.125	0.25	1	1	0.25
	MIC 90	8	4	0.25	1	4	4	2
	Range	0.032–8	0.008–8	0.015–0.5	0.008–1	0.008–8	0.008–8	0.008–8
	*n* (%)	17 (60.7)	5 (17.8)	1 (3.5)	0 (0)	11(39.2)	12(42.8)	3 (10.7)
*S. apiospermum* (*n* = 12)	GM	2.52	1.41	0.35	0.44	1.69	1.20	0.66
	MIC 50	4	1	0.5	0.5	4	1	0.5
	MIC 90	8	8	0.5	1	8	16	8
	Range	0.5–16	0.125–16	0.125–4	0.125–16	0.032–16	0.032–16	0.064–16
	*n* (%)	8 (66.6)	5 (41.6)	1 (8.3)	1 (8.3)	8 (66.6)	5 (41.6)	3 (25)
*S. angustum* (*n* = 5)	GM	2.30	1.15	0.28	0.43	2.30	2	0.32
	Range	1–8	0.25–8	0.064–1	0.064–2	1–4	0.5–4	0.125–0.5
	*n* (%)	3 (60)	2 (40)	0 (0)	1 (20)	4 (80)	4 (80)	0 (0)
*S. aurantiacum* (*n* = 2)	DMic 175378	16	2	0.5	1	8	16	8
	DMic 175588	8	4	0.5	1	4	8	8
	*n* (%)	2 (100)	2 (100)	0 (0)	0 (0)	2 (100)	2 (100)	2 (100)
*S. americanum* sp. nov. (*n* = 2)	DMic 165285	8	1	0.5	1	1	2	0.5
	CBS 128.35	4	0.5	0.25	0.25	2	1	0.25
	*n* (%)	2 (100)	0 (0)	0 (0)	0 (0)	1 (50)	1 (50)	0 (0)
*S. dehoogii* (*n* = 1)	DMic 01867	8	0.25	0.25	0.25	4	4	0.5

AMB: amphotericin B; ITR: itraconazole; VOR: voriconazole; POS: posaconazole; ANI: anidulafungin; CAS: caspofungin; MICA: micafungin. GM: geometric mean; MIC: minimal inhibitory concentration; MEC: minimal effective concentration; * For echinocandins reading was performed at 24 h of incubation and MEC value was considered; *n*/%: number and percentage of species that showed MIC/MEC values ≥2 mg/L.

## Data Availability

The data presented in this study are available in “*Scedosporium* spp. from clinical setting in Argentina, with the proposal of the new pathogenic species *Scedosporium americanum”*.

## Supplementary Materials

Table S1. Preliminary molecular identification of *Scedosporium* Argentinian strains based on *TUB2* sequences. Table S2. Preliminary molecular identification of *Scedosporium* Argentinian strains based on ITS sequences. Table S3. Growing rates of *S. americanum* on OA and PDA at 25 °C.
