# Supplementary material for: Scedosporium spp. from Clinical Setting in Argentina, with the Proposal of the New Pathogenic Species Scedosporium americanum
Source: J Fungi (Basel). 2021 Feb 24;7(3):160. doi: 10.3390/jof7030160 (PMC7995985; doi:10.3390/jof7030160)
Supplement: Supplementary file 1 [file jof-07-00160-s001.pdf]

Supplementary Materials

Table S1. Preliminary molecular identification of *Scedosporium* Argentinian strains based on *TUB2* sequences.

Strain	Blast search ID	Sequence similarity (%)	Query cover	Accession number
DMic 01651	*S. angustum*	99.81	90	AJ889605
DMic 01867	*S. dehoogii*	97.58	100	KX189493
DMic 031321	*S. angustum*	100	90	AJ889605
DMic 062875	*S. boydii*	99.83	97	JQ691004
DMic 073201	*S. boydii*	100	97	JQ690965
DMic 083385	*S. boydii*	100	95	JQ690960
DMic 083389	*S. boydii*	99.83	96	JQ691004
DMic 083404	*S. apiospermum*	100	100	LT558765
DMic 093482	*S. boydii*	99.81	99	JQ691004
DMic 093510	*S. apiospermum*	100	96	JQ690982
DMic 103731	*S. ellipsoideum*	97.5	97	JQ691006
DMic 103813	*S. boydii*	98.09	99	JQ691004
DMic 103859	*S. boydii*	99.24	97	JQ691004
DMic 114031	*S. apiospermum*	100	96	JQ690982
DMic 114032	*S. boydii*	100	95	JQ690988
DMic 114102	*S. angustum*	100	91	AJ889605
DMic 134375	*S. ellipsoideum*	99.3	98	JQ691006
DMic 134638	*S. boydii*	99.83	98	JQ691004
DMic 165033	*S. boydii*	100	95	JQ690965
DMic 165035	*S. apiospermum*	100	100	KY123063
DMic 165036	*S. apiospermum*	100	100	KY123063
DMic 165274	*S. boydii*	99.79	100	KT353440
DMic 165275	*S. angustum*	100	94	AJ889605
DMic 165279	*S. boydii*	98.73	100	JQ690988
DMic 165285	*S. boydii*	86.66	100	JQ691004
DMic 165286	*S. apiospermum*	99.63	100	LT558765
DMic 165288	*S. apiospermum*	100	99	JX032763
DMic 165290	*S. apiospermum*	100	98	JQ690987
DMic 165291	*S. apiospermum*	99.43	100	JQ691003
DMic 165341	*S. boydii*	99.13	99	JQ691004
DMic 175378	*S. aurantiacum*	99	100	JQ690991
DMic 175421	*S. apiospermum*	99.42	100	MG204423
DMic 175525	*S. apiospermum*	98.33	99	JX032763
DMic 175526	*S. boydii*	99.83	95	JQ690960
DMic 175583	*S. boydii*	99.83	96	JQ691004
DMic 175584	*S. angustum*	100	91	AJ889605
DMic 175585	*S. boydii*	100	97	JQ690960
DMic 175588	*S. aurantiacum*	99.83	98	GU126389
DMic 175678	*S. apiospermum*	99.66	98	LT558765
DMic 195868	*S. boydii*	99.83	99	JQ691004
DMic 195869	*S. boydii*	99.63	100	JQ690961
DMic 195871	*S. boydii*	98.97	99	JQ691004
DMic 195874	*S. boydii*	98.93	99	JQ691004
DMic 195875	*S. ellipsoideum*	99.44	98	JQ691006
DMic 195877	*S. boydii*	99.83	98	JQ691004
DMic 195878	*S. boydii*	100	97	JQ690965
DMic 85073	*S. boydii*	99.81	100	JQ690961
DMic 90394	*S. boydii*	99.83	96	JQ691004
DMic 993530	*S. boydii*	99.83	96	JQ691004

Table S2. Preliminary molecular identification of *Scedosporium* Argentinian strains based on ITS sequences.

Strain	Blast search ID	Sequence similarity (%)	Query cover (%)	Accession number
DMic 01651	*S. boydii*	98.37	100	MK637852
DMic 01867	*S. dehoogii*	99.03	100	MT316369
DMic 031321	*S. boydii*	98.41	100	MK637852
DMic 062875	*S. boydii*	99.57	100	MK637852
DMic 073201	*S. boydii*	99.49	100	MK637852
DMic 083385	*S. boydii*	99.57	100	MK637852
DMic 083389	*S. boydii*	99.28	100	MK637852
DMic 083404	*S. apiospermum*	99.46	100	MT316367
DMic 093482	*S. boydii*	99.49	100	MK637852
DMic 093510	*S. apiospermum*	100	100	MT316367
DMic 103731	*S. boydii*	99.28	100	MK637852
DMic 103813	*S. boydii*	99.57	100	MK637852
DMic 103859	*S. boydii*	99.57	100	MK637852
DMic 114031	*S. apiospermum*	100	100	MT316367
DMic 114032	*S. boydii*	99.57	100	MK637852
DMic 114102	*S. boydii*	98.14	98	MK637852
DMic 134375	*S. boydii*	99.31	100	MK637852
DMic 134638	*S. boydii*	99.57	100	MK637852
DMic 165033	*S. boydii*	99.59	99	MK637852
DMic 165035	*S. apiospermum*	99.46	100	MT316367
DMic 165036	*S. apiospermum*	99.32	100	MT316367
DMic 165274	*S. boydii*	99.60	100	MK637852
DMic 165275	*S. boydii*	99.83	99	KC202949
DMic 165279	*S. boydii*	99.34	100	MK637852
DMic 165285	*S. boydii*	99.09	94	AM712309
DMic 165286	*S. apiospermum*	99.58	100	MT316367
DMic 165288	*S. apiospermum*	99.86	100	MT316367
DMic 165290	*S. apiospermum*	99.58	100	MT316367
DMic 165291	*S. apiospermum*	100	100	KT323975
DMic 165341	*S. boydii*	99.54	100	MK637852
DMic 175378	*S. auriantiacum*	100	100	KC254094
DMic 175421	*S. apiospermum*	99.6	100	MH865494
DMic 175525	*S. boydii*	100	100	KM461120
DMic 175526	*S. apiospermum*	99.83	100	MT279297
DMic 175583	*S. boydii*	100	100	KP132699
DMic 175584	*S. boydii*	100	100	KC202949
DMic 175585	*S. boydii*	100	100	MH864722
DMic 175588	*S. auriantiacum*	100	100	LC317769
DMic 175678	*S. apiospermum*	100	100	MT316367
DMic 195868	*S. boydii*	100	100	KM461120
DMic 195869	*S. boydii*	100	100	MH793590
DMic 195871	*S. boydii*	100	100	KM461120
DMic 195874	*S. boydii*	100	99	LC477680
DMic 195875	*S. ellipsoideum*	100	100	JQ690937
DMic 195877	*S. boydii*	100	100	JQ690905
DMic 195878	*S. boydii*	100	100	MH864722
DMic 85073	*S. boydii*	99.42	100	MK637852
DMic 90394	*S. boydii*	99.60	100	MK637852
DMic 993530	*S. boydii*	99.60	100	MK637852

Table S3. Growing rates of *S. americanum* on OA and PDA at 25ºC.

Time (days)	Diameter on OA (mm)		Diameter on PDA (mm)	
	DMic 165285	CBS 218.35	DMic 165285	CBS 218.35
7	18	18	14	15
10	32	27	22	21
14	43	38	30	30
